# A method to estimate the number of people in a country or region with HIV who are undiagnosed and in need of ART

**DOI:** 10.1186/1758-2652-13-S4-P165

**Published:** 2010-11-08

**Authors:** RK Lodwick, CA Sabin, AN Phillips

**Affiliations:** 1 University College London, London, UK

## Purpose of the study

It is important to estimate the number of people in a country who have undiagnosed HIV and low CD4 count as they are in need of ART. New methods are required to derive such estimates.

## Methods

The HIV surveillance data needed are the number of previously undiagnosed people presenting with AIDS (simultaneous HIV/AIDS) in a year, with their CD4 count. The number of people with a simultaneous HIV/AIDS diagnosis in a given CD4 count stratum represents a proportion of the total undiagnosed population with CD4 count in that stratum. This proportion is equivalent to the annual incidence of AIDS in persons in that CD4 count stratum, estimable from cohort studies. For each CD4 count stratum, the number of people with undiagnosed HIV can be estimated by dividing the number of people with simultaneous HIV/AIDS diagnoses (with CD4 count in the stratum) by the CD4-specific AIDS rate. The uncertainty associated with this estimate was evaluated by allowing the AIDS incidence to vary according to a Normal distribution, based on the 95% confidence interval for the incidence rate. The number of simultaneous HIV/AIDS diagnoses was allowed to vary according to a Poisson distribution. These two sources of uncertainty were simultaneously accounted for over 10000 runs.

## Summary of results

The method is illustrated for estimation of the number of people with CD4 count below 200 cells/mm^3^. The incidence rate of AIDS in this CD4 count range has been estimated to be 0.322 (95% CI: 0.268-0.376), using data from CASCADE. Suppose that in the region of interest, in the past year there have been 50 simultaneous HIV/AIDS diagnoses with CD4 count below 200 cells/mm^3^. Then the estimated number of people with undiagnosed HIV with CD4 count below 200 cells/mm^3^ is 156 (as 50/0.322 = 155.3), with a 95% uncertainty range of (109, 213).

## Conclusions

This method allows estimation of the number of people with undiagnosed HIV in any given low CD4 count range, for example below 50 cells/mm^3^ or below 350 cells/mm^3^ (the consensus cut-off used to define a late presenter). These estimates are important for prompting increased efforts to identify people with undiagnosed HIV and low CD4 count, and for planning future delivery of ART.

